# 1,1′,2,2′-Tetra­methyl-3,3′-(*p*-phenyl­ene­dimethyl­ene)diimidazol-1-ium bis­[bis­(trifluoro­methyl­sulfon­yl)imide]

**DOI:** 10.1107/S1600536810038006

**Published:** 2010-09-30

**Authors:** Munirah Sufiyah Abdul Rahim, Yatimah Alias, Seik Weng Ng

**Affiliations:** a Department of Chemistry, University of Malaya, 50603 Kuala Lumpur, Malaysia

## Abstract

The cation of the imidazolium-based ionic-liquid title salt, C_16_H_24_N_4_^2+^·2C_2_F_6_NO_4_S_2_^−^, lies on a center of inversion; in the cation, the five-membered imidazolium ring is aligned at 84.4 (1)° with respect to the phenyl­ene ring; the angle at the methyl­ene C atom is 113.0 (2)°. In the anion, the negative charge formally resides on the two-coordinate N atom; the S—N—S angle at this atom is 125.2 (1)°.

## Related literature

For the tetra­fluoro­borate and hexa­fluoro­phosphate salts, see: Puvaneswary *et al.* (2009*a* ▶,*b* ▶).
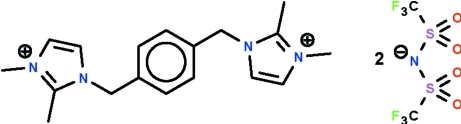


## Experimental

### 

#### Crystal data

C_16_H_24_N_4_^2+^·2C_2_F_6_NO_4_S_2_^−^
*M* *_r_* = 856.71
Monoclinic, 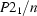

*a* = 8.7195 (7) Å
*b* = 13.710 (1) Å
*c* = 13.8351 (11) Å
β = 92.290 (1)°
*V* = 1652.6 (2) Å^3^
*Z* = 2
Mo *K*α radiation
μ = 0.41 mm^−1^
*T* = 100 K
0.40 × 0.30 × 0.20 mm

#### Data collection

Bruker SMART APEX diffractometer
Absorption correction: multi-scan (*SADABS*; Sheldrick, 1996 ▶) *T*_min_ = 0.853, *T*_max_ = 0.922
10192 measured reflections
3744 independent reflections
3191 reflections with *I* > 2σ(*I*)
*R*_int_ = 0.023

#### Refinement

*R*[*F* ^2^ > 2σ(*F* ^2^)] = 0.034
*wR*(*F* ^2^) = 0.092
*S* = 1.02
3744 reflections
237 parameters
H-atom parameters constrained
Δρ_max_ = 0.48 e Å^−3^
Δρ_min_ = −0.41 e Å^−3^

### 

Data collection: *APEX2* (Bruker, 2009 ▶); cell refinement: *SAINT* (Bruker, 2009 ▶); data reduction: *SAINT*; program(s) used to solve structure: *SHELXS97* (Sheldrick, 2008 ▶); program(s) used to refine structure: *SHELXL97* (Sheldrick, 2008 ▶); molecular graphics: *X-SEED* (Barbour, 2001 ▶); software used to prepare material for publication: *publCIF* (Westrip, 2010 ▶).

## Supplementary Material

Crystal structure: contains datablocks global, I. DOI: 10.1107/S1600536810038006/jh2208sup1.cif

Structure factors: contains datablocks I. DOI: 10.1107/S1600536810038006/jh2208Isup2.hkl

Additional supplementary materials:  crystallographic information; 3D view; checkCIF report

## Figures and Tables

**Fig. 1.**
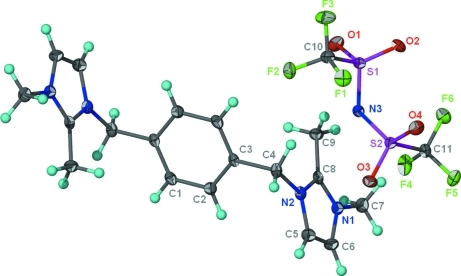
Thermal ellipsoid plot (Barbour, 2001) of C16H24N42+ 2C2F6NO4S2- at the 70% probability level; hydrogen atoms are drawn as spheres of arbitrary radius. Symmetry-related atoms are not labeled.

## supplementary crystallographic information

### Comment

We have previously reported 1,1',2,2'-tetramethyl-3,3'-(*p*-phenylenedimethylene)-bis(imidazol-1-ium) salts (Puvaneswary *et al.*, 2009*a*, 2009*b*). Such compounds are ionic-liquid salts based on an imidazolium entity. The principal feauture of these salts is the non-nucleophilic nature of the counterion. The present bis(trifluoromethanesulfonyl)imide salt (Scheme I, Fig. 1) represents another example of such an anion. The cation lies on a center-of-inversion. The five-membered limidazolyl ring is aligned at with respect to the phenylene ring 84.4 (1) °; the angle at the methylene carbon is 113.0 (2) °. In the anion, the negative charge formally resides on the two-coordinate nitrogen; the angle at this atom is 125.2 (1) °.

### Experimental

1,1',2,2'-Tetramethyl-3,3'-(*p*-phenylenedimethylene)-bis(imidazol-1-ium) bromide (1 mmol) and lithium bis(trifluoromethanesulfonyl)imide (2 mmol) were mixed in water for 2 h to give a solid material. This was collected and recrystallized from ethyl acetate

### Refinement

Carbon-bound H-atoms were placed in calculated positions (C—H 0.95 to 0.99 Å) and were included in the refinement in the riding model approximation, with *U*(H) set to 1.2 to 1.5*U*(C).

### Crystal data

**Table:**

C_16_H_24_N_4_^2+^·2C_2_F_6_NO_4_S_2_^−^	*F*(000) = 868
*M**_r_* = 856.71	*D*_x_ = 1.722 Mg m^−^^3^
Monoclinic, *P*2_1_/*n*	Mo *K*α radiation, λ = 0.71073 Å
Hall symbol: -P 2yn	Cell parameters from 4645 reflections
*a* = 8.7195 (7) Å	θ = 2.7–28.4°
*b* = 13.710 (1) Å	µ = 0.41 mm^−^^1^
*c* = 13.8351 (11) Å	*T* = 100 K
β = 92.290 (1)°	Block, colorless
*V* = 1652.6 (2) Å^3^	0.40 × 0.30 × 0.20 mm
*Z* = 2	

### Data collection

**Table:**

Bruker SMART APEX diffractometer	3744 independent reflections
Radiation source: fine-focus sealed tube	3191 reflections with *I* > 2σ(*I*)
graphite	*R*_int_ = 0.023
ω scans	θ_max_ = 27.5°, θ_min_ = 2.1°
Absorption correction: multi-scan (*SADABS*; Sheldrick, 1996)	*h* = −11→11
*T*_min_ = 0.853, *T*_max_ = 0.922	*k* = −14→17
10192 measured reflections	*l* = −15→17

### Refinement

**Table:**

Refinement on *F*^2^	Primary atom site location: structure-invariant direct methods
Least-squares matrix: full	Secondary atom site location: difference Fourier map
*R*[*F*^2^ > 2σ(*F*^2^)] = 0.034	Hydrogen site location: inferred from neighbouring sites
*wR*(*F*^2^) = 0.092	H-atom parameters constrained
*S* = 1.02	*w* = 1/[σ^2^(*F*_o_^2^) + (0.042*P*)^2^ + 1.3932*P*] where *P* = (*F*_o_^2^ + 2*F*_c_^2^)/3
3744 reflections	(Δ/σ)_max_ = 0.001
237 parameters	Δρ_max_ = 0.48 e Å^−^^3^
0 restraints	Δρ_min_ = −0.40 e Å^−^^3^

### Fractional atomic coordinates and isotropic or equivalent isotropic displacement parameters (Å^2^)

**Table:**

	*x*	*y*	*z*	*U*_iso_*/*U*_eq_	
S1	0.67739 (5)	0.67845 (3)	0.91285 (3)	0.01904 (12)	
S2	0.38200 (5)	0.75917 (3)	0.90601 (3)	0.01821 (12)	
F1	0.71875 (14)	0.85090 (8)	0.83605 (9)	0.0308 (3)	
F2	0.76447 (14)	0.73079 (9)	0.74157 (8)	0.0300 (3)	
F3	0.92506 (13)	0.76619 (10)	0.85879 (10)	0.0390 (3)	
F4	0.21558 (15)	0.60557 (10)	0.94569 (9)	0.0361 (3)	
F5	0.15962 (13)	0.73471 (9)	1.02456 (9)	0.0308 (3)	
F6	0.36490 (15)	0.65461 (10)	1.06344 (8)	0.0347 (3)	
O1	0.73759 (16)	0.58470 (11)	0.89041 (11)	0.0289 (3)	
O2	0.70581 (15)	0.71643 (11)	1.00822 (10)	0.0272 (3)	
O3	0.27597 (15)	0.77674 (11)	0.82658 (10)	0.0277 (3)	
O4	0.43736 (15)	0.83923 (10)	0.96315 (10)	0.0267 (3)	
N1	0.09231 (18)	0.48286 (12)	0.77903 (11)	0.0211 (3)	
N2	0.23507 (18)	0.57396 (12)	0.69354 (11)	0.0208 (3)	
N3	0.50715 (17)	0.68460 (12)	0.87134 (11)	0.0196 (3)	
C1	0.4064 (2)	0.45239 (15)	0.43276 (13)	0.0215 (4)	
H1	0.3417	0.4197	0.3863	0.026*	
C2	0.3415 (2)	0.50636 (15)	0.50513 (13)	0.0219 (4)	
H2	0.2331	0.5103	0.5084	0.026*	
C3	0.4355 (2)	0.55475 (13)	0.57304 (12)	0.0170 (4)	
C4	0.3691 (2)	0.61775 (14)	0.65040 (13)	0.0210 (4)	
H4A	0.4490	0.6297	0.7019	0.025*	
H4B	0.3394	0.6816	0.6219	0.025*	
C5	0.0846 (2)	0.60245 (16)	0.67350 (14)	0.0261 (4)	
H5	0.0510	0.6527	0.6304	0.031*	
C6	−0.0041 (2)	0.54552 (15)	0.72652 (14)	0.0254 (4)	
H6	−0.1128	0.5476	0.7279	0.030*	
C7	0.0391 (2)	0.41044 (15)	0.84840 (14)	0.0242 (4)	
H7A	0.1006	0.4157	0.9090	0.036*	
H7B	0.0503	0.3448	0.8215	0.036*	
H7C	−0.0690	0.4225	0.8610	0.036*	
C8	0.2373 (2)	0.50107 (14)	0.75707 (12)	0.0189 (4)	
C9	0.3763 (2)	0.45077 (15)	0.79565 (14)	0.0239 (4)	
H9A	0.4512	0.4992	0.8198	0.036*	
H9B	0.4210	0.4123	0.7441	0.036*	
H9C	0.3491	0.4075	0.8487	0.036*	
C10	0.7775 (2)	0.76175 (15)	0.83281 (14)	0.0234 (4)	
C11	0.2749 (2)	0.68402 (15)	0.98983 (13)	0.0221 (4)	

### Atomic displacement parameters (Å^2^)

**Table:**

	*U*^11^	*U*^22^	*U*^33^	*U*^12^	*U*^13^	*U*^23^
S1	0.0194 (2)	0.0187 (2)	0.0188 (2)	0.00166 (17)	−0.00064 (16)	0.00181 (17)
S2	0.0183 (2)	0.0185 (2)	0.0177 (2)	0.00097 (16)	−0.00023 (16)	0.00068 (17)
F1	0.0373 (7)	0.0179 (6)	0.0371 (7)	−0.0019 (5)	0.0017 (5)	0.0018 (5)
F2	0.0377 (6)	0.0311 (7)	0.0217 (6)	0.0021 (5)	0.0066 (5)	0.0019 (5)
F3	0.0198 (6)	0.0477 (9)	0.0490 (8)	−0.0072 (5)	−0.0028 (5)	0.0098 (7)
F4	0.0461 (7)	0.0298 (7)	0.0333 (7)	−0.0163 (6)	0.0131 (6)	−0.0077 (5)
F5	0.0296 (6)	0.0330 (7)	0.0306 (6)	0.0035 (5)	0.0110 (5)	−0.0009 (5)
F6	0.0389 (7)	0.0432 (8)	0.0219 (6)	0.0053 (6)	0.0003 (5)	0.0123 (5)
O1	0.0295 (7)	0.0210 (8)	0.0368 (8)	0.0062 (6)	0.0062 (6)	0.0035 (6)
O2	0.0270 (7)	0.0349 (9)	0.0192 (7)	0.0015 (6)	−0.0063 (5)	0.0005 (6)
O3	0.0225 (7)	0.0388 (9)	0.0217 (7)	0.0031 (6)	−0.0024 (5)	0.0091 (6)
O4	0.0260 (7)	0.0191 (7)	0.0349 (8)	−0.0001 (5)	0.0008 (6)	−0.0069 (6)
N1	0.0235 (7)	0.0208 (9)	0.0191 (7)	0.0020 (6)	0.0017 (6)	−0.0006 (6)
N2	0.0248 (8)	0.0203 (9)	0.0173 (7)	0.0009 (6)	0.0026 (6)	0.0001 (6)
N3	0.0191 (7)	0.0207 (9)	0.0189 (7)	−0.0003 (6)	−0.0011 (6)	−0.0047 (6)
C1	0.0241 (9)	0.0226 (10)	0.0177 (8)	−0.0064 (7)	−0.0024 (7)	−0.0027 (7)
C2	0.0194 (8)	0.0249 (10)	0.0214 (9)	−0.0030 (7)	0.0007 (7)	−0.0015 (8)
C3	0.0232 (8)	0.0146 (9)	0.0133 (8)	−0.0031 (7)	0.0015 (6)	0.0026 (6)
C4	0.0275 (9)	0.0180 (10)	0.0177 (8)	−0.0016 (7)	0.0038 (7)	0.0004 (7)
C5	0.0282 (10)	0.0263 (11)	0.0237 (9)	0.0080 (8)	−0.0012 (8)	0.0038 (8)
C6	0.0219 (9)	0.0259 (11)	0.0281 (10)	0.0074 (8)	−0.0027 (7)	−0.0009 (8)
C7	0.0272 (9)	0.0231 (11)	0.0225 (9)	−0.0008 (8)	0.0046 (7)	0.0051 (8)
C8	0.0258 (9)	0.0172 (9)	0.0138 (8)	0.0013 (7)	0.0009 (6)	−0.0014 (7)
C9	0.0218 (9)	0.0235 (10)	0.0260 (10)	0.0039 (7)	−0.0035 (7)	−0.0013 (8)
C10	0.0210 (9)	0.0241 (11)	0.0251 (9)	−0.0002 (7)	0.0001 (7)	0.0030 (8)
C11	0.0249 (9)	0.0234 (10)	0.0182 (9)	−0.0004 (7)	0.0020 (7)	−0.0006 (7)

### Geometric parameters (Å, °)

**Table:**

S1—O1	1.4273 (15)	C1—C2	1.384 (3)
S1—O2	1.4308 (14)	C1—C3^i^	1.387 (2)
S1—N3	1.5724 (15)	C1—H1	0.9500
S1—C10	1.836 (2)	C2—C3	1.390 (3)
S2—O4	1.4255 (14)	C2—H2	0.9500
S2—O3	1.4280 (14)	C3—C1^i^	1.387 (2)
S2—N3	1.5837 (16)	C3—C4	1.509 (2)
S2—C11	1.835 (2)	C4—H4A	0.9900
F1—C10	1.327 (2)	C4—H4B	0.9900
F2—C10	1.332 (2)	C5—C6	1.338 (3)
F3—C10	1.323 (2)	C5—H5	0.9500
F4—C11	1.331 (2)	C6—H6	0.9500
F5—C11	1.327 (2)	C7—H7A	0.9800
F6—C11	1.324 (2)	C7—H7B	0.9800
N1—C8	1.335 (2)	C7—H7C	0.9800
N1—C6	1.387 (2)	C8—C9	1.476 (3)
N1—C7	1.469 (2)	C9—H9A	0.9800
N2—C8	1.330 (2)	C9—H9B	0.9800
N2—C5	1.386 (2)	C9—H9C	0.9800
N2—C4	1.462 (2)		
			
O1—S1—O2	118.48 (9)	C6—C5—N2	106.80 (17)
O1—S1—N3	108.59 (9)	C6—C5—H5	126.6
O2—S1—N3	116.33 (8)	N2—C5—H5	126.6
O1—S1—C10	103.95 (9)	C5—C6—N1	107.28 (17)
O2—S1—C10	105.12 (9)	C5—C6—H6	126.4
N3—S1—C10	102.19 (9)	N1—C6—H6	126.4
O4—S2—O3	119.43 (9)	N1—C7—H7A	109.5
O4—S2—N3	116.29 (8)	N1—C7—H7B	109.5
O3—S2—N3	107.98 (8)	H7A—C7—H7B	109.5
O4—S2—C11	104.56 (9)	N1—C7—H7C	109.5
O3—S2—C11	104.53 (9)	H7A—C7—H7C	109.5
N3—S2—C11	101.73 (9)	H7B—C7—H7C	109.5
C8—N1—C6	108.92 (16)	N2—C8—N1	107.58 (16)
C8—N1—C7	126.98 (16)	N2—C8—C9	125.43 (17)
C6—N1—C7	124.10 (16)	N1—C8—C9	126.99 (17)
C8—N2—C5	109.41 (16)	C8—C9—H9A	109.5
C8—N2—C4	125.98 (16)	C8—C9—H9B	109.5
C5—N2—C4	124.60 (16)	H9A—C9—H9B	109.5
S1—N3—S2	125.16 (10)	C8—C9—H9C	109.5
C2—C1—C3^i^	121.01 (17)	H9A—C9—H9C	109.5
C2—C1—H1	119.5	H9B—C9—H9C	109.5
C3^i^—C1—H1	119.5	F3—C10—F1	108.73 (17)
C1—C2—C3	119.81 (17)	F3—C10—F2	108.50 (16)
C1—C2—H2	120.1	F1—C10—F2	107.88 (16)
C3—C2—H2	120.1	F3—C10—S1	110.21 (13)
C1^i^—C3—C2	119.18 (17)	F1—C10—S1	111.04 (13)
C1^i^—C3—C4	119.47 (16)	F2—C10—S1	110.41 (14)
C2—C3—C4	121.31 (16)	F6—C11—F5	108.38 (15)
N2—C4—C3	112.97 (15)	F6—C11—F4	108.32 (17)
N2—C4—H4A	109.0	F5—C11—F4	107.65 (15)
C3—C4—H4A	109.0	F6—C11—S2	110.87 (13)
N2—C4—H4B	109.0	F5—C11—S2	110.29 (13)
C3—C4—H4B	109.0	F4—C11—S2	111.22 (12)
H4A—C4—H4B	107.8		
			
O1—S1—N3—S2	159.88 (11)	C6—N1—C8—N2	0.8 (2)
O2—S1—N3—S2	23.18 (16)	C7—N1—C8—N2	−178.01 (17)
C10—S1—N3—S2	−90.68 (13)	C6—N1—C8—C9	−179.42 (18)
O4—S2—N3—S1	14.34 (16)	C7—N1—C8—C9	1.7 (3)
O3—S2—N3—S1	151.77 (12)	O1—S1—C10—F3	−67.76 (16)
C11—S2—N3—S1	−98.55 (13)	O2—S1—C10—F3	57.41 (16)
C3^i^—C1—C2—C3	0.3 (3)	N3—S1—C10—F3	179.31 (14)
C1—C2—C3—C1^i^	−0.3 (3)	O1—S1—C10—F1	171.70 (13)
C1—C2—C3—C4	177.36 (17)	O2—S1—C10—F1	−63.13 (15)
C8—N2—C4—C3	75.5 (2)	N3—S1—C10—F1	58.76 (15)
C5—N2—C4—C3	−104.1 (2)	O1—S1—C10—F2	52.09 (15)
C1^i^—C3—C4—N2	−140.06 (17)	O2—S1—C10—F2	177.26 (13)
C2—C3—C4—N2	42.3 (2)	N3—S1—C10—F2	−60.84 (15)
C8—N2—C5—C6	0.2 (2)	O4—S2—C11—F6	−59.50 (15)
C4—N2—C5—C6	179.92 (17)	O3—S2—C11—F6	174.21 (13)
N2—C5—C6—N1	0.3 (2)	N3—S2—C11—F6	61.92 (15)
C8—N1—C6—C5	−0.7 (2)	O4—S2—C11—F5	60.54 (15)
C7—N1—C6—C5	178.19 (18)	O3—S2—C11—F5	−65.75 (15)
C5—N2—C8—N1	−0.7 (2)	N3—S2—C11—F5	−178.04 (13)
C4—N2—C8—N1	179.66 (16)	O4—S2—C11—F4	179.90 (13)
C5—N2—C8—C9	179.58 (18)	O3—S2—C11—F4	53.62 (16)
C4—N2—C8—C9	−0.1 (3)	N3—S2—C11—F4	−58.68 (15)

Symmetry codes: (i) −*x*+1, −*y*+1, −*z*+1.
